# Hypogonadotropic Hypogonadism in a Central Obesity Patient with 46, XY, t(1; 10)(q42; q24)

**DOI:** 10.5935/1518-0557.20250039

**Published:** 2025

**Authors:** Han Luo, Hui Cao, Jiehan Zhang, Qiao Zhang, Huiqing Li, Lulu Chen, Tianshu Zeng, Xiang Hu

**Affiliations:** 1 Department of Endocrinology, Union Hospital, Tongji Medical College, Huazhong University of Science and Technology, Wuhan 430022, China; 2 Hubei Provincial Clinical Research Center for Diabetes and Metabolic Disorders, Wuhan 430022, China; 3 Hukou County People’s Hospital, Jiujiang 332500, China; 4 Department of Cardiovascular Surgery, Union Hospital, Tongji Medical College, Huazhong University of Science and Technology, Wuhan 430022, China; † The authors contributed equally to this case report

**Keywords:** Hypogonadism, Obesity, Chromosomal Abnormalities, Chromosomal Translocation

## Abstract

Hypogonadotropic hypogonadism (HH) is characterized by absent or insufficient secretion of GnRH and/or pituitary gonadotropins, which consequently leads to testicular dysfunction. HH is commonly associated with obesity, particularly in specific genetic syndromes, and its etiology appears heterogeneous and remains far from fully understood. This report describes the case of a 31-year-old male with HH and central obesity, who has a karyotype of 46, XY, t(1; 10)(q42; q24). He demonstrated a good pituitary response to GnRH as well as a robust testicular reserve in both the GnRH stimulation test and the hCG stimulation test. Notably, he exhibited no lifestyle risk factors for obesity or comorbidities likely contributing to his condition. The SCD1 gene, located at 10q24.31, promotes the synthesis of fatty acids, and its abnormality may play a significant role in the pathogenesis of obesity. We propose that the translocation between chromosome 1q42 and chromosome 10q24 may disrupt the function of SCD1 at chromosome 10q24, resulting in the mutually reinforcing relationship between obesity and HH in this patient.

## INTRODUCTION

Male hypogonadotropic hypogonadism (HH) is considered a consequence of congenital or acquired diseases that affect the hypothalamus and/or the pituitary gland. It is characterized by absent or inadequate secretion of gonadotropin-releasing hormone (GnRH) and/or pituitary gonadotropins, which consequently induces testicular dysfunction, impairing spermatogenesis and/or testosterone production (Fraietta *et al.*, 2013). Numerous studies indicate that congenital hypogonadotropic hypogonadism (CHH) is clinically and genetically heterogeneous. More than 40 genes have been identified to play essential roles in the development, migration, and function of GnRH neurons, which are closely related to CHH (Butz *et al.*, 2021).

In recent years, an increasing number of studies have shown that obesity is closely linked to lower free testosterone concentrations in male adolescents and adults. An extensive study conducted in the United States found that 40% of obese men had subnormal free testosterone levels (Dhindsa *et al.*, 2010). Furthermore, it has been reported that the prevalence of HH among 160 obese men (mean age 58 years) is 36% in a study from the Netherlands (Dhindsa *et al.*, 2018). Additionally, HH coincides with obesity in specific genetic syndromes, such as Prader-Willi syndrome, Bardet-Biedl syndrome, and Blakemore-Durmaz-Vasileiou syndrome, which are associated with complex disorders, including but not limited to growth hormone deficiency, cognitive impairment, neurodevelopmental delay, and rod-cone dystrophy (Bosch *et al.*, 2021; Iughetti *et al.*, 2019; Sloboda *et al.*, 2022). The etiology of HH and obesity is not entirely clear. This article describes the case of a male with HH and obesity, with 46, XY, t(1; 10)(q42; q24) karyotype.

## CASE REPORT

### Case Presentation

The patient was a 31-year-old male, weighing 86 kg, with a height of 180 cm and an abdominal circumference of 90 cm. His pubic hair development was rated Tanner stage 4, while his penis and testicular development were rated Tanner stage 3. He had been obese since childhood without overfeeding and had experienced erectile dysfunction, diminished libido, and sparse pubic hair since puberty. His testosterone levels were low, and he used chorionic gonadotropin for three years, during which his secondary sexual characteristics developed and his erectile function improved. After discontinuing chorionic gonadotropin treatment, the symptoms of erectile dysfunction, diminished libido, and sparse pubic hair worsened. He denied any family history of obesity or abnormal sexual development but reported a history of hyperlipidemia and hyperuricemia for 5 years and osteoporosis for 2 years. Additionally, he underwent radiofrequency catheter ablation for supraventricular tachycardia at the age of 17.

Further testing indicated that his testosterone level was 7.21 nmol/L (reference range 8.6-29.0 nmol/L), which was associated with anomalous luteinizing hormone (2.92 IU/L) and follicle-stimulating hormone (6.62 IU/L) levels, without any apparent abnormalities in adrenal and thyroid function. Therefore, he was diagnosed with obesity-related hypogonadotropic hypogonadism (Saboor Aftab *et al.*, 2013), and treated with lifestyle interventions combined with orlistat at a dosage of 0.12 g three times daily. His body weight gradually decreased, and blood testosterone levels improved to 12.6 nmol/L after the first month and 18.6 nmol/L after three months.

### Laboratory tests

The patient’s testosterone level was 7.21 nmol/L (Table 1), which is below the reference range. No significant abnormalities were noted in the complete blood count, routine urine analysis, serum electrolytes, cortisol and thyroid hormone levels, or liver and kidney function tests.

**Table 1:** Hormone analysis.

Hormone	Patient valve	Normal range	units
LH	2.92	1.7-8.6	IU/L
FSH	6.62	1.5-12.4	IU/L
Progesterone	0.05	<0.149	ng/ml
Estradiol	38.43	11.3-43.2	pg/ml
PRL	19.18	3.86-22.8	ng/ml
Testosterone	7.21	8.6-29.0	nmol/l
SHBG	9.70	18.3-54.1	nmol/l
DHEA-S	13.19	4.34-12.2	umol/L
Androstenedione	2.54	0.51-3.02	ng/ml

### GnRH stimulation test

**Figure 1:**
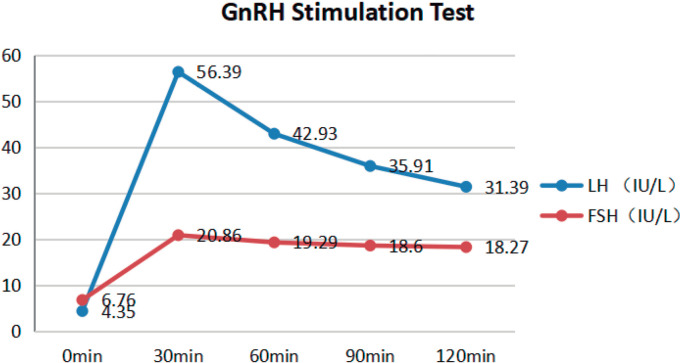
The GnRH stimulation test. LH and FSH levels peaked 30 minutes after the administration of Gonadorelin, reaching values of 56.39 IU/L and 20.86 IU/L, respectively.

A GnRH stimulation test was performed between 8:00 AM and 10:00 AM after at least 8 hours of overnight fasting. Gonadorelin^®^ (BBCA Pharmaceutical, Anhui, China) was administered intravenously at a dosage of 100 mg. Venous blood samples were collected at 0 (before the administration of Gonadorelin), and at 15, 30, 60, and 120 minutes after the injection to assess the levels of luteinizing hormone (LH) and follicle-stimulating hormone (FSH) (Mosbah *et al.*, 2020). The results indicated peaks of LH and FSH of 56.39 IU/L and 20.86 IU/L, respectively (Figure 1).

### Human chorionic gonadotropin (hCG) stimulation test

**Figure 2:**
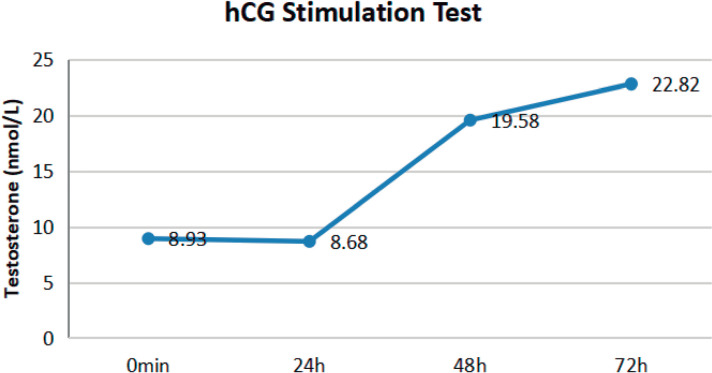
The hCG stimulation test. The testosterone level peaked at 72 hours after the hCG injection, reaching 22.82 nmol/L.

The hCG stimulation test was performed using an intramuscular injection of 2000 U hCG over three consecutive days, with blood samples collected before the first dose of hCG (0 minutes) and again after 24, 48, and 72 hours (Lucas-Herald *et al.*, 2020). The peak testosterone level was 22.82 nmol/L following 72 hours of hCG injection (Figure 2).

### Oral glucose tolerance test

**Figure 3:**
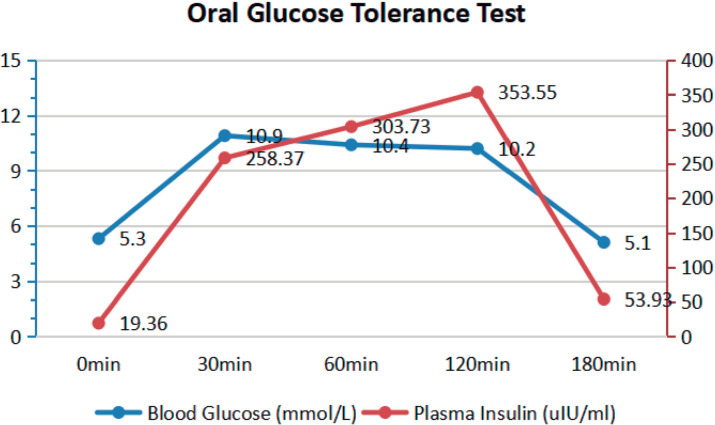
Oral glucose tolerance test. The 2-hour post-load plasma glucose was 10.2 mmol/L, and the peak insulin level was 353.55 uIU/ml, which occurred at the 2-hour post-load plasma glucose measurement.

The oral glucose tolerance test was conducted using a 75-g glucose load after an overnight fast. Blood samples were collected before the test and at 30, 60, 120, and 180 minutes, with measurements taken for glucose and insulin levels (Wang *et al.*, 2018). The results indicated that the plasma glucose level was 5.3 mmol/L before the administration of glucose. In comparison, the 2-hour post-load plasma glucose level was 10.2 mmol/L, surpassing the upper limit of normal plasma glucose (less than 7.8 mmol/L) after 2 hours, indicating that the patient had impaired glucose tolerance (Bartoli *et al.*, 2011). His insulin level was 258.37 IU/mL at 30 minutes after glucose loading, peaking at 120 minutes (353.55 IU/mL), which was over 18 times higher than the baseline level (19.36 IU/mL); it remained at 53.93 IU/mL at 180 minutes, implying impairments in first-phase insulin secretion and increased late-phase insulin secretion (Figure 3).

### Scrotal ultrasonography

**Figure 4:**
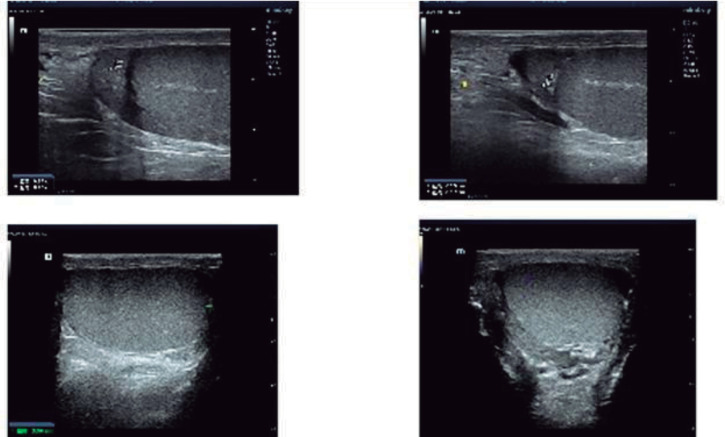
Scrotal ultrasonography. Both testicles were located in the scrotum. The right testicle measured 33.6×29.8×19.1 mm, while the left measured 36.6×25.1×19.8 mm. Each head of the epididymis contained a cyst (1.5×1.2 mm on the right side, 1.4×1.3 mm on the left side).

Scrotal ultrasonography revealed the right testicle size to be 33.6×29.8×19.1 mm and the left testicle size to be 36.6×25.1×19.8 mm, which appeared within the normal reference range (Lotti *et al.*, 2021). Each head of the epididymis had a cyst (1.5×1.2 mm on the right side, 1.4×1.3 mm on the left side) (Figure 4).

### Chromosome analysis

**Figure 5:**
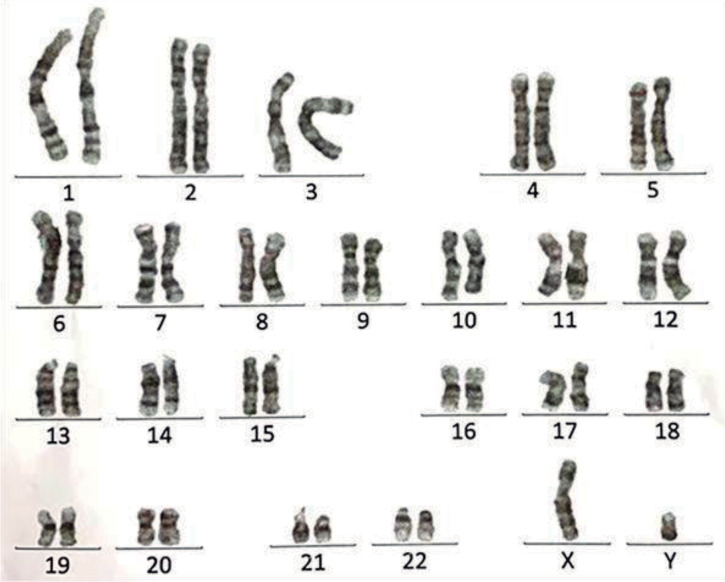
Karyotyping analysis. G-band karyotyping indicated that the patient’s karyotype is 46, XY, t(1;10)(q42;q24), suggesting a translocation between chromosome 1q42 and chromosome 10q24 in the patient.

G-band karyotyping analysis revealed that the patient’s karyotype was 46, XY, t(1;10)(q42;q24), indicating a translocation between chromosome 1q42 and chromosome 10q24 (Figure 5).

### Genetic sequencing

The patient’s exome and mitochondrial genome sequencing data were analyzed against the OMIM database (2021Q3). No significant pathogenic mutations were identified in association with the clinical phenotype.

## DISCUSSION

This article describes the case of a 31-year-old male with central obesity and HH with 46, XY, t(1; 10)(q42; q24). The patient did not experience diseases likely to cause obesity, such as hypothyroidism, Cushing syndrome, and others. He did not show lifestyle risk factors for obesity, such as overeating. Notably, the patient suffered from hypogonadism, which is widely considered one of the significant causative factors in the etiology of obesity (Genchi *et al.*, 2022). Hypogonadism may play a crucial role in the pathogenesis of obesity.

Our results indicated that the patient had no other conditions, such as Prader-Willi or Bardet-Biedl syndrome, that might cause or be associated with obesity. Interestingly, the results of the GnRH stimulation test revealed that the peak FSH was more than 12 times higher, and the peak LH was more than three times higher than the baseline levels (Figure 1), suggesting that the patient had a strong pituitary response to GnRH (Ab Rahim *et al.*, 2020). Moreover, the results of the hCG stimulation test showed that the peak testosterone level was over two times higher than the baseline level (Figure 2), implying that the patient possessed a substantial testicular reserve (Bang *et al.*, 2017). All these clinical features are closely related to obesity-related hypogonadism (Saboor Aftab *et al.*, 2013). Additionally, it is widely acknowledged that HH and obesity create a vicious cycle, in which obesity can lead to HH, and HH, in turn, promotes obesity (Carrageta *et al.*, 2019). Thus, we tentatively propose that the HH was likely attributed to obesity in this patient.

Karyotype analysis revealed a translocation between chromosomes 1q42 and 10q24. The *stearoyl-CoA desaturase 1* (SCD1) gene, located at 10q24.31, promotes the synthesis of fatty acids and plays a crucial role in maintaining energy homeostasis. Dysfunction of SCD1 is a significant factor in obesity (Pan *et al.*, 2021). Therefore, the translocation between chromosome 1q42 and chromosome 10q24 may result in an abnormal chromosome 10q24, subsequently leading to SCD1 dysfunction, which contributes to the development of obesity.

There is a lack of familial data to confirm the association between the chromosomal translocation of chromosomes 1q42 and 10q24, obesity, and HH. We were unable to identify an alteration in the SCD1 gene in the patient; however, the dysregulation of SCD1 likely mediated the obesity and HH stemming from the chromosomal translocation involving chromosomes 1q42 and 10q24. Additionally, we were unable to develop an animal model with the translocation to demonstrate the causative role of the chromosomal translocation described above in the pathogenesis of obesity and HH. Therefore, further family investigations and/or animal studies are necessary to verify the association among the chromosomal translocation, obesity, and HH.

Despite these limitations, we tentatively propose that the translocation between chromosome 1q42 and chromosome 10q24 is closely associated with obesity and HH, although further confirmatory research is needed. It seems reasonable to suggest that more attention should be given to chromosome abnormalities in obese patients with hypogonadism.

## Data Availability

The original contributions presented in the study are included in the article and supplementary material. Any further inquiries can be directed to the corresponding author.
